# Enhanced vertical second harmonic generation from layered GaSe coupled to photonic crystal circular Bragg resonators

**DOI:** 10.1515/nanoph-2024-0282

**Published:** 2024-08-05

**Authors:** Zhuojun Liu, Bo Chen, Xuying Wang, Guixin Qiu, Qitao Cao, Dunzhao Wei, Jin Liu

**Affiliations:** State Key Laboratory for Mesoscopic Physics and Frontiers Science Center for Nano-Optoelectronics, School of Physics, 12465 Peking University, Beijing 100871, China; State Key Laboratory of Optoelectronic Materials and Technologies, School of Physics, School of Electronics and Information Technology, Sun Yat-Sen University, Guangzhou 510275, China

**Keywords:** two-dimensional layered materials, circular Bragg gratings, second harmonic generation

## Abstract

Two-dimensional (2D) layered materials without centrosymmetry, such as GaSe, have emerged as promising novel optical materials due to large second-order nonlinear susceptibilities. However, their nonlinear responses are severely limited by the short interaction between the 2D materials and light, which should be improved by coupling them with photonic structures with strong field confinement. Here, we theoretically design photonic crystal circular Bragg gratings (CBG) based on hole gratings with a quality factor as high as *Q* = 8 × 10^3^, a mode volume as small as *V* = 1.18 (*λ*/*n*)^3^, and vertical emission of light field in silicon nitride thin film platform. Experimentally, we achieved a *Q* value up to nearly 4 × 10^3^, resulting in a 1,200-fold enhancement of second harmonic generation from GaSe flakes with a thickness of 50 nm coupling to the CBG structures under continuous-wave excitation. Our work endows silicon-based photonic platforms with significant second-order nonlinear effect, which is potentially applied in on-chip quantum light sources and nonlinear frequency conversion.

## 1 Introduction

Second harmonic generation (SHG), which converts two incident photons into one radiation photon with doubled frequency, is commonly used to characterize second-order nonlinearities of materials. Conventional bulk materials, such as beta barium borate, potassium dihydrogen phosphate and lithium niobate, have shown efficient SHG via phase matching or quasi-phase matching, facilitating the development of tunable laser source, nonlinear beam shaping, and generation of quantum entanglement states [1], [2], [3], [4]. In recent years, the production of thin-film platforms with second-order nonlinear coefficients (*χ* ^(2)^), such as lithium niobate, aluminium nitride, indium phosphide, have boosted research on integrated nonlinear photonic devices, featuring scalability, reliability, and low power consumption [5], [6], [7], [8]. However, integrating these emerging nonlinear platforms with CMOS-compatible platforms remains challenging, hindering the fabrication of large-scale photonic circuits with *χ* ^(2)^ effects [9], [10].

Two-dimensional (2D) materials have become a focal point in nonlinear photonics due to their unique properties, including their thin layered structure, ease of hybrid integration through van der Waals forces, and large second-order nonlinearities [11]. Few-layered structures of Hexagonal boron nitride, black phosphorus and transition metal dichalcogenides exhibit significant SHG response, but the counteraction of SHG from adjacent layers makes the increase in the number of layers ineffective, severely limiting the conversion efficiencies of SHG [12], [13], [14], [15]. Noncentrosymmetric layered materials ranging from monolayer to bulk, such as *ϵ*-GaSe and NbOI_2_, are another promising class of 2D nonlinear materials. The second-order nonlinear response of these materials increases with their thickness within one coherent length [14]. Additionally, their *χ* ^(2)^ could reach as large as ∼1,000pm/V , nearly two orders of magnitude larger than that of conventional bulk nonlinear crystals [16], [17]. Noncentrosymmetric layered materials with huge *χ* ^(2)^ enable the production of notably nonlinear processes, which is highly desirable for constructing integrated nonlinear devices for optical frequency conversion and generation of quantum light sources [18], [19], [20], [21]. Nevertheless, the limited interaction length remains a bottleneck for producing notable SHG under excitation of a continuum-wave (CW) laser [22].

Integrating 2D materials with photonic structures has been widely used to address the challenge of enhancing second-order nonlinearities. Materials with huge *χ* ^(2)^ are often accompanied by strong absorption, making increasing the interaction length under the phase matching scheme a suboptimal way to increase SHG conversion efficiency [23]. Instead, the use of microcavities is preferred due to their smaller size, facilitating alignment with mechanically stripped high-quality 2D materials, and their ability to strongly confine the field, thereby enhancing light–matter interaction [24], [25], [26]. Different kinds of microcavities, such as microdisk, photonic crystal slabs based on bound states in the continuum, and photonic crystal defect cavities, with 2D materials attached to them, have shown enhanced SHG due to their high-*Q* factor and small mode volume. The dependence of second harmonic power on the square of the *Q* factor of the pump beam enables to produce notably SHG even under excitation of CW lasers. However, attaching thicker 2D layered materials onto these cavities encounters degradation of the *Q* factor due to the sensitivity of cavity resonance to the surrounding environment [27], [28], [29], [30]. A circular Bragg grating (CBG) structure, also known as bullseye cavity, featuring a lager Purcell factor and vertical emission of Gaussian-like field, have been employed to couple with quantum dots or 2D materials for efficiently generating fluorescence emission, quantum sources, and nonlinear waves [31], [32]. The vertical emission field of the CBG structure can sufficiently interact with covering 2D materials and facilitate the collection of signal fields. However, most reported CBG structures used concentric ring gratings, struggling to increase the *Q* value, especially for thin-film platforms like silicon nitride (SiN) with a relatively smaller refractive index. Recently, CBG structures based on hole gratings have shown a much higher *Q* factor with a nearly unchanged mode volume, i.e. a larger Purcell factor [33], [34], but employing the second-order Bragg condition imposes limitation on further improving the *Q* factor.

Here, we demonstrate a large enhancement of SHG in layered GaSe flakes with 50 nm in thickness by coupling them with an optimally designed resonant CBG structure based on hole gratings and the first-order Bragg condition. Using simulations based on the finite-difference time-domain method (FDTD), we present strong confinement of optical field in the optimized CBG structure with a simulated *Q* factor up to 8 × 10^3^ and predominantly vertical emission of the resonant field. Furthermore, the SHG from the GaSe flakes is enhanced by 1,200 times by integrating them with the fabricated CBG structure under CW excitation. This enhancement benefits from an almost unaffected *Q* factor in the hybrid integration, reaching up to nearly 4 × 10^3^. Our work provides an effective way to promote the nonlinear optical performance of thicker 2D layered materials, which is very advantageous for the development of practical nonlinear and quantum light sources.

## 2 Device design

**Figure 1:**
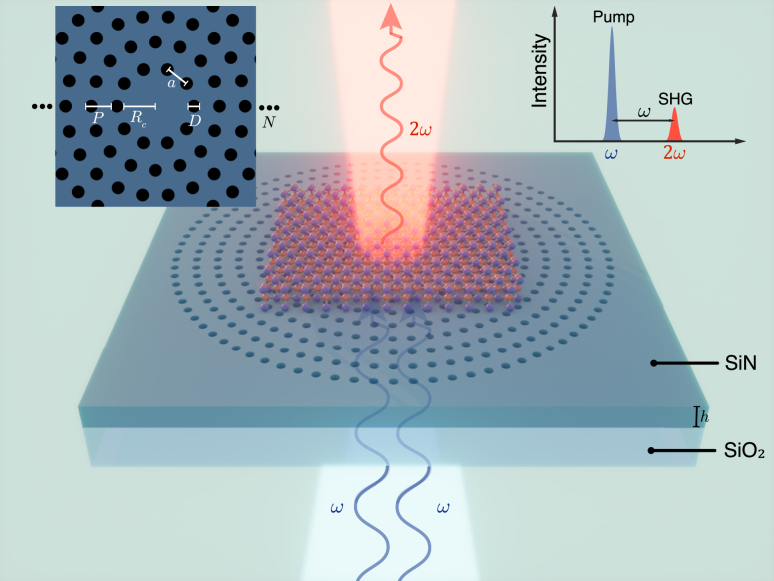
Conceptual diagram of SHG from the hybrid integrated device composed of CBG structure and GaSe flakes. Inset is top view of the designed CBG structure with definition of structural parameters.

Figure 1 shows the conceptual diagram of the hybrid integrated device composed of 2D layered GaSe flakes adhered to a SiN CBG structure, along with the SHG processes. The CBG structure comprises two main regions: a central disk confining a resonant mode and surrounding annular periodic hole gratings that acts as lateral reflective mirrors. A CW laser, serving as the pump beam, is normally incident on the device to excite the resonant mode at the fundamental frequency and then produce second harmonic signal from the GaSe flake in the transmission direction. The interaction between the resonant mode and the GaSe flakes converts two photons at a lower frequency into one photon at a high frequency, following the conservation law of energy. To realize a high-performance device, the parameters of CBG should be carefully optimized, including the thickness *h* of the SiN layer, the radius *R* of the central disk, the diameter *D* of the hole, the radial periodicity *P* and azimuthal periodicity *a* of the surrounding hole gratings, and the number *N* of annular hole gratings, as shown in the inset of Figure 1. The equal azimuthal periodicity of each ring ensures consistent effective refractive index along the radial direction.

**Figure 2:**
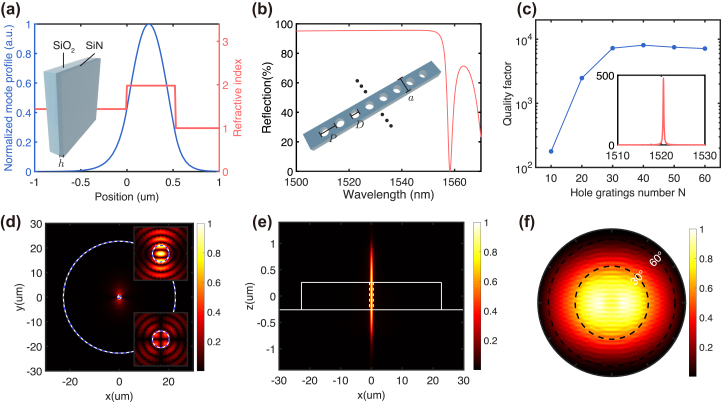
The designed CBG structure (a) TE fundamental mode profile in the one-dimensional SiN slab waveguide. (b) Calculated reflection band of one-dimensional first-order Bragg hole gratings with parameters of *D* = 130 nm, *P* = 440 nm, and *a* = 250 nm. (c) Simulated *Q* factors dependence on the number *N* of hole gratings for the whole CBG structure. The inset shows the calculated Purcell factors when *R* = 625 nm. (d) On-resonance near-field intensity distribution at *XY*-plane with *X* = 0. Right upper and lower insets show the intensity distributions of *E*_*x*_ and *E*_*y*_ components, respectively. (e) On-resonant intensity distribution at *XZ*-plane with *Y* = 0. (f) On-resonance far-field intensity pattern at *XY*-plane.

We first performed one-dimensional simulation on a SiN slab to determine its thickness *h* that supports only the transverse electric (TE) and transverse magnetic (TM) fundamental modes. Figure 2(a) shows the TE fundamental mode profile along the vertical direction in a 520 nm thick SiN slab. Then, we used a row of circular holes, instead of slots, along the radial direction to carry out primitive but rapid simulation and optimization of the CBG structure, as shown in the inset of Figure 2(b). In this simplified three-dimensional simulation, a TE mode is incident from one side of the hole gratings to obtain the reflection spectrum. By adjusting the parameters *a*, *D*, and *P* of the one-dimensional hole gratings to satisfy the first-order Bragg reflection condition, we achieved a broadband high reflection by suppressing lateral leakage, as shown in Figure 2(b). Using these parameters, we further investigated the optical properties of the CBG structure, in which an *X*-polarized electric dipole source was placed in the central disk to excite the resonant modes. Because the hole gratings play a key role in lateral confinement, the *Q* factor depends highly on the number *N*, as presented in Figure 2(c). It increases with *N* and saturates at *N* > 40, reaching a maximum of *Q* = 8,000. The simulated *Q* factor is at least one orders of magnitude higher than the previous CBG structure using ring gratings as lateral reflectors in the same platform, indicating the potential for SHG increasement by two orders of magnitude [35], [36]. This improvement is attributed to the simultaneous optimization of structure parameters in both the radial and azimuthal directions, effectively suppressing higher-order diffraction. The inset in Figure 2(c) shows a clear resonance peak centered at ∼1,520 nm with a calculated Purcell factor of approximately 480 when the central disk radius *R* is 625 nm. The resonant wavelength is determined by the following equation [37]:

(1) $$n_{\text{disk}}\times 2R+n_{\text{grating}}\times 2L_{\text{eff}}=m\times \lambda .$$

Here, *n*_disk_ is an effective refractive index in the central disk, *n*_grating_ is the effective index in the area of hole gratings, *L*_eff_ is the effective length in hole gratings, all pertaining to the mode index *m*. The Purcell factor is calculated as the ratio of the radiation output from a dipole in the structure to that from a dipole in vacuum, demonstrating the enhancement of spontaneous emission into the cavity mode. After performing a parametric study to optimize the *Q* factor for the targeted resonant wavelength of 1,520 nm, we obtained the optimized design parameters of the CBG structure as follows: *h* = 520 nm, *R* = 625 nm, *D* = 130 nm, *P* = 440 nm, *a* = 250 nm, and *N* = 50. The value of *P* = 440 nm indicates the utilization of the first-order Bragg condition. The designed CBG structure possesses 180° rotational symmetry rather than circular symmetry, leading to a polarization-dependent resonant wavelength and corresponding electric field distribution. Here we focused on the resonant mode with dominated *X*-polarization, which is excited by a source with the same polarization state in the simulation. This resonant mode features tight mode confinement in the central disk (as seen in the *XY*-plane field profile in Figure 2(d)) and highly directional emission with a relatively small divergence angle (as seen in *XZ*-plane field profile in Figure 2(e) and *XY*-plane far-field patterns in Figure 2(f)). According to Figure 2(d) and (e), the mode volume is calculated to be *V* = 1.18 (*λ*/*n*)^3^, resulting in a Purcell factor of 480. Notably, the electric field distribution localized at the upper surface of the CBG structure is strong enough to promote the nonlinear effect of the GaSe flakes. This is because the surrounding hole gratings provide strong lateral confinement to boost the vertical emission.

## 3 Results and discussion

**Figure 3:**
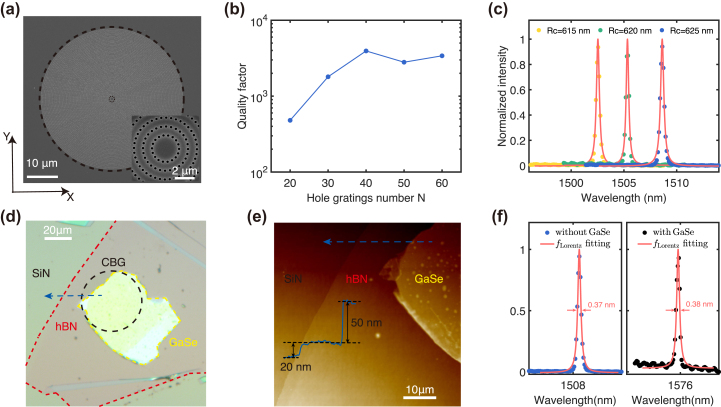
The fabricated CBG structure (a) SEM image of the fabricated CBG structure. Scale bar: 1 μm. (b) The dependence of *Q* factors on the number *N* of hole gratings. (c) The measured resonant scattering spectrum of the CBG structures with different radii of the central disk. (d) Optical microscope image of the hybrid integrated device. Scale bar: 10 μm. (e) AFM image of the boundary of GaSe flake (red frame in (d)). Scale bar: 5 μm. The inset shows that the measured thickness of the GaSe flakes is 50 nm along the bule arrow line. (f) The scattering spectrum of the CBG structure before and after the integration of the GaSe flakes and the layered hBN.

The designed CBG structure was fabricated in a 520 nm thick SiN layer on a 7 mm thick quartz substrate by using electron beam lithography and induced coupled plasma etching process. Figure 3(a) shows the scanning electron microscope (SEM) image of the fabricated CBG structure. It shows well-distributed hole gratings surrounding a central disk. Subsequently, we used the resonant scattering spectrum to characterize the resonant wavelength of the *X*-polarized resonant mode and measured the *Q* factor with different numbers *N* of hole gratings, as shown in Figure 3(b). The *Q* factor exhibits an increasing trend as *N* increases, which becomes saturated at *N* > 40, reaching a maximum *Q* factor of up to 4,000. It is well matched with the simulated results depicted in Figure 2(c). Additionally, Figure 3(c) show the scattering spectrum with different central disk radii of the CBG structure (*R* = 615 nm, 620 nm, 625 nm). A sharp peak was observed at 1,508 nm when *R* was 625 nm. Clearly, the resonant wavelength can be continuously adjusted, with a blueshift trend as the central disk radius decreases, which is very agreement with Eq. (1). Next, we mechanically exfoliated a 2D layered GaSe sample from a bulk GaSe material and then attached it onto the CBG structure with *R* = 625 nm using a dry transfer technique [38]. Then a 20 nm thick hBN layer was used to encapsulated the GaSe flakes, protecting it from degradation in the air environment [39]. Figure 3(d) displays the optical microscope image of the hybrid integrated device. The GaSe layer with a sufficient large area covers the whole the central disk in the CBG structure without requiring a high-precision alignment procedure, enabling interaction between the whole resonant mode and the GaSe flakes for efficient nonlinearities. As seen from Figure 3(e), the thickness of the GaSe layer was measured to be 50 nm by using an atomic force microscope (AFM), corresponding to 56 layers when considering the monolayer thickness of 0.89 nm. To further characterize the influence of the GaSe flakes on the resonance of the CBG structure, the resonant scattering spectra of the CBG structure before and after the integration of the GaSe flakes are shown in Figure 3f. A redshift of approximately 67 nm for the resonant wavelength is observed owing to the large thickness of the GaSe flakes, but the robust resonant mode makes the *Q* factor unchanged. The maintaining high *Q* factor presents that the CBG structure is well-suited for integration with relatively thick 2D materials to develop high-performance second-order nonlinear optical devices.

**Figure 4:**
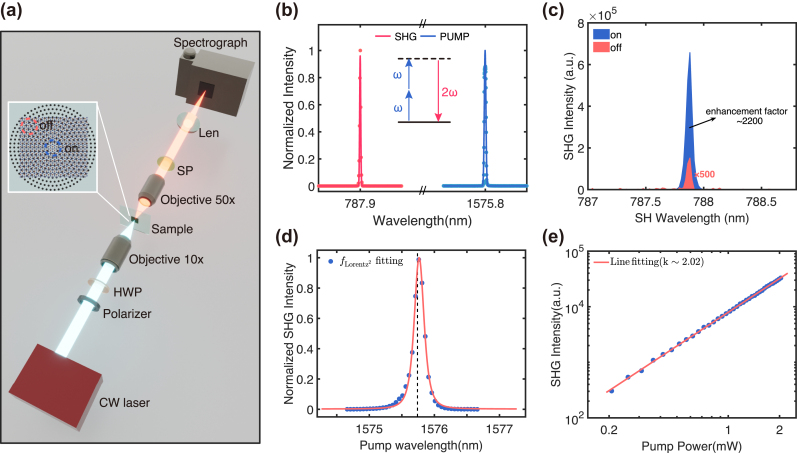
The nonlinear performance of the hybrid integrated devices (a) Illustration of the optical setup for SHG characterization. SPF, short-pass filter with a cut-off wavelength of 1,000 nm. CW, continuous wave; P, polarizer; HWP, half-wave plate. (b) Normalized on-resonance spectra of pump beam (right) and second harmonic signal (left) generated from the hybrid integrated device. (c) Measured spectra of the SHG from GaSe flakes on (blue line) and off (red line) the CBG structure under the on-resonance wavelength of 1,575.8 nm. (d) Measured intensities of SHG when scanning the pump wavelength with the Lorentzian fitting. (e) Measured SHG intensities as a function of the pump power using a double logarithmic plot. The exponent value obtained from the fitting is 1.973.

The SHG process was used to characterized the nonlinear performance of the hybrid integrated devices, as illustrated in Figure 4(a). A CW laser with tunable wavelength from 1,480 nm to 1,620 nm served as the pump source. It was focused to a 3 μm-in-diameter spot via a 20× objective lens (NA = 0.45) in order to match the size of the central disk, so that the incident power can sufficiently couple into the resonant mode. A polarizer and a half wave plate were used to control the linear polarization state along the *X*-axis. The output SHG signal along the transmission direction was collected by a 50× objective lens (NA = 0.65) and then sent into a spectrometer (PISP 2758) after the pump laser beam was removed by a 1,000 nm short-pass filter.

Figure 4(b) shows typical measured spectra of the pump beam (blue line) and the corresponding second harmonic signal (red line). A clear signal appears at 787.9 nm when the pump laser operates at the resonant wavelength of 1,575.8 nm, providing evidence of frequency doubling in the SHG process. Figure 4(c) shows the observed enhancement of about 2,000 times for SHG from the covering GaSe flakes on the CBG structure (blue line) compared to that from the GaSe flakes off the CBG structure (red line). The inset in Figure 4(a) shows the positions of on and off the CBG for SHG measurement. This SHG enhancement is comparable to that from GaSe flakes attached on high-*Q* cavities [27], [38], [40] and significantly superior to that from CBG structures based on ring gratings [35]. It could be further improved by optimizing the size of the pump beam to more efficiently couple its energy into the cavity. It should be noted that the SHG response from the hBN layer in the device is negligible due to its much smaller second-order susceptibility of ∼42pm/V compared to that of GaSe flakes.

To confirm the critical role of SHG under the resonant excitation, we performed dependence of second harmonic intensity on the pump wavelength, as shown in Figure 4(d). When scanning the pump laser across the resonant mode, the SHG changes drastically and reaches a maximum when the pump laser is on resonance. Conversely, when the pump laser is detuned from the resonant wavelength, the SHG becomes weak, indicating that such an enhancement arises from the coupling between the GaSe flakes and the CBG structure. The resonant curve of SHG in Figure 4(d) can be strictly fitted with the squared Lorentzian function, which is attributed to the Lorentzian shape of the fundamental resonant mode. Figure 4(e) further presents the experimental second harmonic intensity as a function of pump power using a double logarithmic plot. The slope value of the linear fitted line (red curve) is close to the theoretical value of 2, which is commonly believed to be a signature of the SHG process. A second harmonic power of 10.42 pW was detected using an incident pump power of 2 mW, giving a measured efficiency of 2.60 × 10^−6^ /W for the hybrid device. The SHG response of GaSe flakes belonging to $\bar{6}$ 2*m* point group is generally independent on the incident polarization [18], while the 180° rotational symmetry of the CBG structure results in a polarization-dependent resonant wavelength. Therefore, to maintain the second harmonic efficiency of our devices, the pump wavelength should be readjusted to be on-resonance when changing the polarization states.

## 4 Conclusions

We have successfully achieved enhanced SHG from the GaSe flakes integrated on the optimally designed photonic crystal CBG structure based on hole gratings. The CBG structure has undergone thorough numerical investigation and optimization, obtaining a quality factor as high as *Q* = 8 × 10^3^ and a mode volume as small as *V* = 1.18 (*λ*/*n*)^3^. Additionally, it facilitates pronounced vertical emission of the resonant field, a characteristic commonly observed in second-order Bragg gratings. The experimental *Q* value of the hybrid integrated device is up to 4 × 10^3^, nearly the same as the raw CBG structures, resulting in a second harmonic efficiency of 2.60 × 10^−6^ /W with 2,000-fold enhancement. This efficient SHG is attributed to the combination of the high *Q* factor and small mode volume as well as the strong *χ* ^(2)^ of the thicker layered GaSe flakes. Our work offers crucial guidance for the utilization of 2D materials in conjunction with the photonic structures and provides avenues for the development of high-performance on-chip nonlinear applications in frequency conversion, quantum light sources, and beyond [41].
